# Understanding and mitigating the environmental barriers that patients with major depressive disorder face when transitioning to independent exercise

**DOI:** 10.1177/13591045211024226

**Published:** 2021-06-12

**Authors:** Nilanga Aki Bandara

**Affiliations:** Department of Kinesiology, 8166 The University of British Columbia, Vancouver, BC, Canada

Jarbin et al.’s (2021) study highlight the potential benefits of a group exercise program for young patients with persistent major depression. When evaluating the exercise program’s impact on depressive symptoms in the long-term, there was a statistically significant (*p* < .001) reduction in scores of both the Quick Inventory of Depressive Symptomatology (QIDS-A) clinician-administered and self-reported versions. Further, when comparing baseline to one-year follow-up, the metric used to measure wellbeing found a statistically significant (*p* = .011) improvement in participants’ wellbeing. However, the authors share an important consideration involving the challenges associated with transitioning from group-based exercise programs to independently directed exercise. This challenge can limit participation in exercise, thus hindering the benefits of exercise. One of the potential reasons for this observed challenge could be due to environmental barriers associated with exercise, including the distance to gyms and gym costs (Glowacki et al., 2017; Monteiro et al., 2020). These environmental barriers can make it quite challenging for young people with persistent depression to exercise independently.

Distance to the gym was a barrier noted by Monteiro et al. (2020), in this study 53.2% of participants either agreed or strongly agreed that distance to the gym was a barrier to exercise. Other studies in the literature have found that the distance to physical activity resources, such as gyms, are negatively correlated with physical activity (Jilcott et al., 2007). Further, given the age of adolescents, they may not have access to a driver’s license or vehicle for transportation to physical activity spaces that are not accessible by public transportation. One way to mitigate the barrier of gym distance is to provide patients transitioning from group-based to individual exercise programs with home gym equipment and virtual instruction resources. This intervention is even more appealing given our current context living amidst the Covid-19 pandemic, where access to many physical activity resources have been limited. By engaging in physical activity at home with sufficient equipment, the issue of distance to physical activity resources is greatly reduced. Further, by supplementing equipment with virtual instruction, we are providing continued support for patients to engage in physical activity. Virtual instruction could be provided by a kinesiologist or a personal trainer through a convenient online platform.

Both Monteiro et al. (2020) and Glowacki et al. (2017) found that the costs associated with physical activity acted as a barrier to physical activity engagement. Adolescents may not have the financial means to purchase a gym membership and they may have to rely on their parents for financial support. Further, it can be expected that physical activity costs can be even more prohibitive for young patients coming from low socioeconomic status (SES) families, as they may already be dealing with financial struggles. Thus, physical activity may not be financially attainable for patients transitioning from group-based to independently led exercise. In order to mitigate this challenge, providing free access to physical activity resources can reduce the financial constraints associated with physical activity. To put this into action, vouchers could be given to adolescents, so they can access physical activity resources. Higgerson et al. (2018) evaluated the effectiveness of free access to community fitness spaces and outreach on physical activity levels, it was found that the physical activity levels of the population were increased, while also reducing the inequalities associated with accessing physical activity resources for people with low SES. Additionally, the idea of providing home gym equipment alongside virtual instruction can also reduce the costs associated with exercising at other physical activity spaces.

Ultimately, the environmental challenges associated with physical activity for adolescent patients with major depression can make it difficult for them to exercise. Providing home gym equipment alongside virtual instruction and/or subsidizing physical activity resources can reduce the impact of these environmental barriers. Moving forward, implementation and evaluation of these proposed strategies can provide further guidance on ways to support young patients with major depression to exercise.
